# Classifying domain-specific text documents containing ambiguous keywords

**DOI:** 10.1093/database/baab062

**Published:** 2021-11-26

**Authors:** Kamran Karimi, Sergei Agalakov, Cheryl A Telmer, Thomas R Beatman, Troy J Pells, Bradley I M Arshinoff, Carolyn J Ku, Saoirse Foley, Veronica F Hinman, Charles A Ettensohn, Peter D Vize

**Affiliations:** Department of Biological Sciences, University of Calgary, Calgary, AB T2N 1N4, Canada; Department of Biological Sciences, University of Calgary, Calgary, AB T2N 1N4, Canada; Department of Biological Sciences, Carnegie Mellon University, Pittsburgh, PA 15213, USA; Department of Biological Sciences, Carnegie Mellon University, Pittsburgh, PA 15213, USA; Department of Biological Sciences, University of Calgary, Calgary, AB T2N 1N4, Canada; Department of Biological Sciences, University of Calgary, Calgary, AB T2N 1N4, Canada; Department of Biological Sciences, Carnegie Mellon University, Pittsburgh, PA 15213, USA; Department of Biological Sciences, Carnegie Mellon University, Pittsburgh, PA 15213, USA; Department of Biological Sciences, Carnegie Mellon University, Pittsburgh, PA 15213, USA; Department of Biological Sciences, Carnegie Mellon University, Pittsburgh, PA 15213, USA; Department of Biological Sciences, University of Calgary, Calgary, AB T2N 1N4, Canada

## Abstract

A keyword-based search of comprehensive databases such as PubMed may return irrelevant papers, especially if the keywords are used in multiple fields of study. In such cases, domain experts (curators) need to verify the results and remove the irrelevant articles. Automating this filtering process will save time, but it has to be done well enough to ensure few relevant papers are rejected and few irrelevant papers are accepted. A good solution would be fast, work with the limited amount of data freely available (full paper body may be missing), handle ambiguous keywords and be as domain-neutral as possible. In this paper, we evaluate a number of classification algorithms for identifying a domain-specific set of papers about echinoderm species and show that the resulting tool satisfies most of the abovementioned requirements. Echinoderms consist of a number of very different organisms, including brittle stars, sea stars (starfish), sea urchins and sea cucumbers. While their taxonomic identifiers are specific, the common names are used in many other contexts, creating ambiguity and making a keyword search prone to error. We try classifiers using Linear, Naïve Bayes, Nearest Neighbor, Tree, SVM, Bagging, AdaBoost and Neural Network learning models and compare their performance. We show how effective the resulting classifiers are in filtering irrelevant articles returned from PubMed. The methodology used is more dependent on the good selection of training data and is a practical solution that can be applied to other fields of study facing similar challenges.

Database URL The code and date reported in this paper are freely available at http://xenbaseturbofrog.org/pub/Text-Topic-Classifier/

## Introduction

Text mining in biological data has been of much interest (1–3), including in improving biocuration work (4, 5). Given the amount of textual data available online, and sometimes the urgent nature of searching through them (6, 7), classifying (8) text based on certain topics is a major task in many fields. Resources such as NCBI’s PubMed provide keyword-based searches to help researchers obtain papers of interest. This is much easier than searching all available articles, but PubMed’s returns may contain irrelevant papers if the keywords apply to multiple domains. Traditionally, deciding whether a paper returned by keyword search is relevant for a field of study has required an expert to read the paper and make a decision, known as triage. This can be a time-consuming curation process, hence the efforts to automate it (5), including in our context, which is model organism databases (9, 10). In this paper, we apply a number of general text classification methods to the problem of filtering PubMed returns for articles on echinoderm biology (11) and develop a set of tools to perform the filtering. The phylum Echinodermata consists of a very diverse group of species such as starfish, sea urchins, brittle stars, feather stars and sea cucumbers.

Echinobase (www.echinobase.org) (12), the online knowledgebase for echinoderms, is tasked with curating genomic information from relevant papers. As can be guessed from the common names, a keyword search for such varied species produces a large number of false-positive returns. We encountered this problem when retrieving papers from the PubMed database with keywords search using E-Utilities (13). Our aim was to download as many relevant papers as possible. The search parameters used individual terms (‘sea’ and ‘urchin’ vs. ‘sea urchin’) and the search also looked at the body of the papers. The returns included most of the relevant papers but also many irrelevant ones. For example, the word ‘sea’ appears in many irrelevant cases. The false-positive matches in Echinobase include papers on astronomy (stars), botany (cucumber), surgery (scars the shape of a starfish), environmental sciences (sea star–like plastic floating in the water) and other unrelated fields of study.

The initial approach was for the Echinobase curation team to inspect the downloaded papers and reject the irrelevant ones. Our keyword search returned over 18 000 papers from PubMed, and the curation team, after verifying a subset of them, estimated that between 30% and 50% were not relevant or not of interest to Echinobase users. Other possible solutions to reducing the number of returned papers include limiting the search to titles and abstracts only and searching for multi-word keywords when possible, which reduce the number of irrelevant returns but do not filter all of them. As a policy, Echinobase has decided to err on the side of not missing relevant papers, and given the time-consuming nature of manually identifying false positives, we developed the automated classification system described in this paper, to help the curation process.

As shown in ref. (14), text mining using the full body of papers generally gives better results. Full text, if available, can be incorporated into the training sets and used in the proposed solution. However, because of copyright restrictions, many papers only have their title and abstract available, so we use these two fields to classify them. Having the full text does provide more information, but in our experience, most authors provide the important keywords in the title and abstract. As a result, we do not consider the lack of full text to be a major limiting factor. At the same time, not depending on the presence of full body increases the applicability of the solution.

## The method

Relying on only titles and abstracts both limits the amount of information (undesirable) and focuses on the parts with the main message of the papers (desirable). As with any supervised learning effort, having an appropriate mix of relevant and irrelevant papers as training data is the key to achieving good classification accuracy. We tried to solve the ambiguity problem through careful selection of training papers, making sure common species names appear often enough to be learned by the classifiers. We note that all domain-dependent parts of this work are encapsulated in the training data, and the following methodology is domain-neutral.

We used Python to develop the code and used classifiers offered by the Scikit-learn (sklearn) machine learning library (15). For each paper in the training and testing sets, we concatenated the title and the abstract, converted them to a bag of words and computed the relative frequency of each word’s occurrence using sklearn’s TfidfVectorizer. The output is sparse matrices representing the relative frequency of words used in the abstracts and titles. The results were fed into a number of classifiers. The main criteria when selecting the classifiers to use were their popularity as well as their ability to process sparse data. Table 1 lists the evaluated classifiers and notes their underlying classification model. We note that RandomForest, Bagging and AdaBoost are ensemble classifiers. With both Bagging and AdaBoost, we used the default decision tree as the base classifier, so all three ensemble classifiers were based on decision trees.

**Table 1.** Sklearn classifiers used in our experiments

Classifiers	Model
RidgeClassifier, SGDClassifier, PassiveAggressiveClassifier, LogisticRegression	Linear
MultinomialNB, ComplementNB, BernoulliNB	Naïve Bayes
DecisionTreeClassifier, RandomForestClassifier, BaggingClassifier, AdaBoostClassifier	Tree
KNeighborsClassifier	K Nearest Neighbors
SVC	SVM
MLPClassifier	Neural Network

We used accuracy, precision and recall to measure the performance of the classifiers. Scikit-learn provides methods to calculate and report these metrics, which we employed. We developed command line tools for training and testing the classifiers and for running the classifiers on one or more text files.

All code and data needed to run the tool and reproduce the results are freely available at http://xenbaseturbofrog.org/pub/Text-Topic-Classifier/. The code was developed and tested with Python 3.7.4. The package includes code for training and testing the classifiers, a tool for running the classifiers on individual articles, as well as a tool for running the classifiers on multiple articles in batch mode. These tools allow the readers to incorporate this filtering mechanism into their existing literature loader pipelines: An existing software system to download papers from a resource such as PubMed can pass the contents of downloaded papers to the tools and decide to add or reject the papers based on the classifier’s output.

All computations reported in the next section were performed on a PC with an Intel i7-8700 CPU with 12 logical (hyper-threaded) cores and 32 GB of RAM. The time it takes to train a classifier varies with the type of classifier, with ensemble ones being the slowest, as expected. When possible, we instructed Scikit-learn to use all available cores. During the training phase, CPU usage was mostly at 100% for ensemble classifiers, with the PC being very sluggish, and around 60–80% for other classifiers. The maximum RAM usage during the training phase was around 4 GB, with 1 GB being the usual usage. Training, excluding the optional cross-validation phase, took less than 5 seconds for all the classifiers except Bagging and MLPClassifier, which took around a minute each. Please note that even small differences in training times are amplified when doing a large number of cross validations (CVs). Classification times mirror the training times. As an example, the Bagging classifier, using all available cores, took about 30 seconds to classify the 248 papers mentioned below, while the Ridge classifier took less than 2 seconds to process the same files.

## Evaluation

 We used the results of our PubMed E-Utilities keyword search for echinoderms, which contained numerous relevant and irrelevant papers, to create training and testing sets. Professional curators manually selected a set of 496 relevant (positive) papers and a set of 405 irrelevant (negative) papers to train the classifiers. We tried to keep the selected papers, both relevant and irrelevant, as varied as possible, so care was taken for the relevant set to contain most of the echinoderm species of interest in Echinobase, though due to their high number, not all were covered.

To test the classifiers, 229 relevant and 220 irrelevant papers were selected by the curators. We performed 20-fold CV on the training dataset to get a measure of their suitability, then fitted the data to the training set and finally tried the resulting classifiers on the test dataset. The results appear in Table 2, where average CV accuracy and standard deviation values for the training data and the accuracy values for the test data are shown.

**Table 2.** Accuracy values for the training and test data. Best accuracies are highlighted in bold

	20-fold CV for training data		
Classifier	Average accuracy (%)	Standard deviation	Accuracy for test data (%)
RidgeClassifier	**88.4**	4.8	88.4
SGDClassifier	88.2	5.1	88.6
PassiveAggressiveClassifier	88.0	4.7	89.3
LogisticRegression	87.4	5.6	86.6
MultinomialNB	82.4	7.9	79.5
ComplementNB	84.1	7.0	82.0
BernoulliNB	82.1	7.6	83.7
DecisionTreeClassifier	86.4	5.9	92.7
RandomForestClassifier	87.4	6.0	88.0
BaggingClassifier	87.5	5.1	**95.3**
KNeighborsClassifier	82.1	5.4	81.1
AdaBoostClassifier	84.0	6.1	91.1
SVC	88.0	4.5	89.1
MLPClassifier	87.1	5.7	88.2

While most classifiers achieve similar results for training and test accuracy, Decision Tree, AdaBoost and Bagging perform much better on the test data. In general, we expect the results to be data- and domain-dependent, so we encourage the readers to try as many of the classifiers as possible on their own data. We also note that results may change with fine-tuning classifier parameters. The exact parameters used by us are in the source code.

Since accuracy does not provide a complete picture, we also measured precision, recall and F-score values for the test data, as shown in Table 3. These measures are important because they allow us to know, for both relevant and irrelevant classes, what percentage of the relevant, or irrelevant, papers were assigned the correct class (precision) and what percentage of each class were correctly retrieved (recall). F-score, which combines the precision and recall scores, can be used as a single number to select the most suitable method. In some contexts, it may be important to not miss any relevant papers, at the expense of having some irrelevant ones added to the accepted list. In other contexts, it may be preferable to filter out more of the irrelevant papers, even if that leads to misclassifying some relevant ones. This is why we report precision and recall values (vs. only the F-score) for both relevant and irrelevant papers. The results allow us to see the unbalanced filtering of some of the classifiers, which make them unsuitable for our task. We note that based on precision and recall values, Bagging provides the best results for our data.

**Table 3.** Precision and recall values for the test dataset. Best results are highlighted in bold

	Irrelevant papers			Relevant papers		
Classifier	Precision (%)	Recall (%)	F-score (%)	Precision (%)	Recall (%)	F-score (%)
RidgeClassifier	91.6	84.1	87.7	85.8	92.6	89.1
SGDClassifier	91.6	84.5	87.9	86.2	92.6	89.3
PassiveAggressiveClassifier	91.7	78.6	85.2	82.1	94.3	87.8
LogisticRegression	93.0	78.6	85.2	82.1	94.3	87.8
MultinomialNB	95.7	60.9	74.4	72.2	97.4	82.9
ComplementNB	92.6	68.6	78.9	75.9	94.8	84.3
BernoulliNB	96.2	69.5	80.7	76.9	97.4	85.9
DecisionTreeClassifier	93.9	91.5	92.4	90.9	94.3	92.9
RandomForestClassifier	95.1	79.5	86.5	83.0	96.1	89.1
BaggingClassifier	**96.3**	**94.1**	**95.2**	**94.4**	**96.5**	**95.5**
KNeighborsClassifier	89.0	70.0	78.4	76.1	91.7	83.2
AdaBoostClassifier	94.1	87.3	90.6	88.6	94.8	91.6
SVC	88.8	86.8	87.8	87.6	89.5	88.6
MLPClassifier	94.9	84.1	89.2	86.2	95.6	90.7

In another test, we measured the ability of the Bagging classifier to reject irrelevant papers returned from PubMed. To do so, we collected 248 papers from PubMed which were returned when searching for keywords in the titles, abstracts and bodies but not when excluding the bodies. We expect these papers to contain ambiguous uses of our keywords and hence be mostly irrelevant. A domain expert reviewed the papers and determined 242 of them to be indeed irrelevant and 6 to be relevant. The classifier declared 240 papers as being irrelevant and 8 as relevant. The classifier and the domain expert agreed on 5 out of the 6 relevant papers and 239 out of 242 irrelevant papers, resulting in a 98.3% accuracy (4 mistakes out of 248). We consider the results to confirm that leaving out the full body in echinoderm paper search from PubMed will greatly reduce the number of irrelevant returns, although it is possible to lose relevant papers.

We compared our test accuracy results with NCBI’s LitSuggest (16), an online system that works similarly to ours. It has a web-driven graphical interface that makes it easy to use. However, that means it cannot be programmatically integrated into any literature download pipelines. LitSuggest can only work with PubMed papers, while our system can use any set of text files as input. This is not an obstacle in a comparison test because all of our datasets were sourced from PubMed. We trained a LitSuggest classifier using our training data and then ran it on our test data. The resulting accuracy was at 92.8% (vs. Bagging’s 95.3%). For the set of 248 test papers, LitSuggest made 12 mistakes (it agreed with the domain expert in four out of six relevant cases), resulting in an accuracy of 95.1% (vs. Bagging’s 98.3%). We attribute most of these differences in performance to LitSuggest’s choice of classifiers.

## Conclusions and future work

Our problem involves sorting relevant and irrelevant publications in the presence of ambiguous keywords. One possible line of action is to remove some of the irrelevant search returns by excluding the paper body and look for multi-word keywords as much as possible but that increases the probability of missing relevant papers. An alternative approach is to cast a wide search net and retrieve more papers from resources such as PubMed and then use classifiers to reject the irrelevant ones. To do so, we evaluated a variety of classification methods with very different underlying algorithms. In our case, we chose the Bagging method based on high accuracy, as well as balanced precision and recall values.

We consider the training data to be the key factor in obtaining our results. A number of different curators, over a course of few months, were involved in creating the training and test data. These were professional biologists with varying degrees of familiarity with echinoderms. During the development phase, we noticed that some papers were classified as both relevant and irrelevant. We also noted that in a few cases the same paper switched from relevant to irrelevant or vice versa over time. We attribute these to the fact that different curators may have different opinions about what papers should be included in Echinobase. We also expect the database’s priorities and focus to change over time. This means that improving the classifiers with different or better training data is a continuing process. Over time, clearly miss-classified papers can be added to the relevant and irrelevant training sets, to hopefully achieve better results with retrained classifiers.

It is important to note that while we used specific domain knowledge (echinoderms) to create the classifiers, the underlying software design is domain-neutral, and the same methodology can be applied in other fields. Given this data dependency, we encourage the readers to try all or most of these classification algorithms when solving similar problems in other domains and choose the better ones. If a number of classifiers show clear advantages over others, they can be combined in an ensemble classifier, such as sklearn’s VotingClassifier, to hopefully improve the results further.

Established model organism databases such as Xenbase (17) focus on a smaller number of species, so filtering irrelevant papers is not a big problem. However, they may curate papers with a wider variety of contents, including gene expression, diseases, phenotypes and a variety of ontologies such as Gene Ontology (GO) (http://geneontology.org/). Considering the encouraging results of this paper, we intend to extend the work and use it to perform multi-class tagging, where papers downloaded from PubMed are classified as containing different topics of interest. Such a classification system will speed up the paper curation process.
